# Flexible Film Bulk Acoustic Resonator Based on Low-Porosity β-Phase P(VDF-TrFE) Film for Human Vital Signs Monitoring

**DOI:** 10.3390/s23042136

**Published:** 2023-02-14

**Authors:** Zhentao Yu, Feng Gao, Xiangyu He, Hao Jin, Shurong Dong, Zhen Cao, Jikui Luo

**Affiliations:** 1 Key Laboratory of Advanced Micro/Nano Electronic Devices and Smart Systems of Zhejiang, College of Information Science and Electronic Engineering, Zhejiang University, Hangzhou 310027, China; 2 ZJU-Hangzhou Global Scientific and Technological Innovation Center, Hangzhou 311215, China

**Keywords:** P(VDF-TrFE), film bulk acoustic resonator, flexible pressure sensor, vital signs monitoring

## Abstract

P(VDF-TrFE) is a promising material for flexible acoustic devices owing to its good piezoelectric performance and excellent stretchability. However, the high density of internal pores and large surface roughness of the conventional P(VDF-TrFE) results in a high propagation attenuation for acoustic waves, which limits its use in flexible acoustic devices. In this paper, a novel method based on two-step annealing is proposed to effectively remove the pores inside the P(VDF-TrFE) film and reduce its surface roughness. The obtained P(VDF-TrFE) film possesses excellent characteristics, including a high breakdown strength of >300 kV/mm, a high-purity β-phase content of more than 80%, and high piezoelectric coefficients (d_33_) of 42 pm/V. Based on the low-porosity β-phase P(VDF-TrFE) film, we fabricated flexible film bulk acoustic resonators (FBARs) which exhibit high sharp resonance peaks. The pressure sensor was made by sandwiching the FBARs with two PDMS microneedle patches. Heartbeat and respiration rate monitoring were achieved using the pressure sensor. This work demonstrates the feasibility of high-performance flexible piezoelectric acoustic resonators based on low-porosity P(VDF-TrFE) films, which could see wider applications in the wearable sensors for both physical and chemical sensing.

## 1. Introduction

Flexible and wearable sensors advanced rapidly in recent decades due to their promising potential in personal healthcare applications [1]. Film bulk acoustic resonators (FBARs) have been widely used in conventional solid-state sensors for the detection of both physical and chemical parameters due to their high frequency sensitivity, such as UV intensity [2], pressure [3], temperature [4], humidity [5], gas [6], etc. FBAR is made of a floating piezoelectric film sandwiched by two metal electrodes, whose resonance frequency varies with external environmental variations [7]. Nevertheless, due to the difficulties in the fabrication of high-quality flexible piezoelectric films, there are few applications of flexible FBARs in wearable devices. The selection of piezoelectric materials is one of the most critical issues for the fabrication of high-performance FBAR devices. P(VDF-TrFE) is a promising candidate for the fabrication of wearable FBAR sensors [8] due to its good piezoelectric characteristics, inherent flexibility, biocompatibility, and low cost [9]. Although P(VDF-TrFE) has been widely used in devices such as hydroacoustic transducers [10], ultrasonic detection sensors [11], acceleration sensors [12], and energy harvesters [13], there are few reports on the application of P(VDF-TrFE) in the field of FBAR, due to its porous structure, crystal defects, and rough surface. The operation of FBAR relies on the standing acoustic wave resonance, due to the reflection between the two air-film boundaries [14]. When acoustic waves propagate in a porous film, refraction and reflection occur due to the existence of pores and defects, which results in a high acoustic attenuation and even device failure. Therefore, the preparation of low-porosity and high-crystallinity P(VDF-TrFE) films is the key for making high-performance flexible FBARs.

Various methods have been reported for improving the performance of P(VDF-TrFE) films. One of the solutions is to use inorganic nanofillers to make composite materials with PVDF. The inorganic nanofiller can be PZT [15], BaTiO_3_ nanoparticles (NPs) [16], ZnO nanoparticles [17], porous cellulose nanofibrils (CNFs) [18], etc. However, the addition of nanofillers creates additional defects at the interface between the filler and the polymer [19]. Even if the two materials are closely fitted, the acoustic impedance mismatch between them will degrade the performance of the fabricated acoustic devices. Increasing the proportion of β-phase PVDF in the composite film by stretching can also improve the piezoelectricity of the film because β-phase has the highest piezoelectric response among all the crystalline phases of PVDF. In addition to stretching, it is also reported that the proportion of β-phase can be increased by changing the annealing temperature of recrystallization during fabrication [20]. The annealing temperature is usually set between the Curie temperature (110 °C) and the melting point (152 °C) [21]. Despite the significant impact of the porosity on the performance of FBARs made from P(VDF-TrFE), little progress has been made in reducing it because of its flocculent crystal structure and the required high-temperature annealing process that results in a high susceptibility to pores and cracks.

The existing pressure sensors can also achieve heartbeat and respiratory monitoring; for example, capacitive pressure sensors using porous PDMS micro-pillars as a dielectric layer [22], piezoresistive sensors based on graphene-PDMS @ sponge [23], and pressure sensors based on P(VDF-TrFE)/BaTiO_3_ nanocomposite micropillar nanogenerators [16]. Nevertheless, these sensors still have various drawbacks. For instance, capacitive sensors suffer from poor linearity, while nanogenerators can only respond to pulsed signals. In contrast, the P(VDF-TrFE) FBAR-based flexible pressure sensor has the advantages of good linearity, simple fabrication process, and low cost. Moreover, the materials used to prepare the devices in this paper are all biocompatible, which is perfect for wearable devices.

In this paper, we report the fabrication of high-performance flexible FBARs based on low-porosity β-phase P(VDF-TrFE) films. The β-phase P(VDF-TrFE) film is prepared by a novel process with a recrystallization annealing temperature higher than the melting point of P(VDF-TrFE). The flowability of P(VDF-TrFE) above the melting point allows for the efficient removal of internal pores under the vacuum and reduces the surface roughness, which results in the high β-phase and excellent piezoelectric properties of the P(VDF-TrFE) film. Due to the high quality of the P(VDF-TrFE) film, the resonance characteristics of the FBARs made with this film are significantly enhanced compared with those made with ordinary P(VDF-TrFE) films. Using the P(VDF-TrFE) FBAR, a flexible pressure sensor is fabricated, which has a resolution of 1 kPa and exhibits excellent response speeds. Heartbeat and respiration rate monitoring is successfully demonstrated using the flexible FBAR sensor.

## 2. Materials and Methods

### 2.1. Preparation of the Low-Porosity β-Phase P(VDF-TrFE) Film

**Figure 1:**
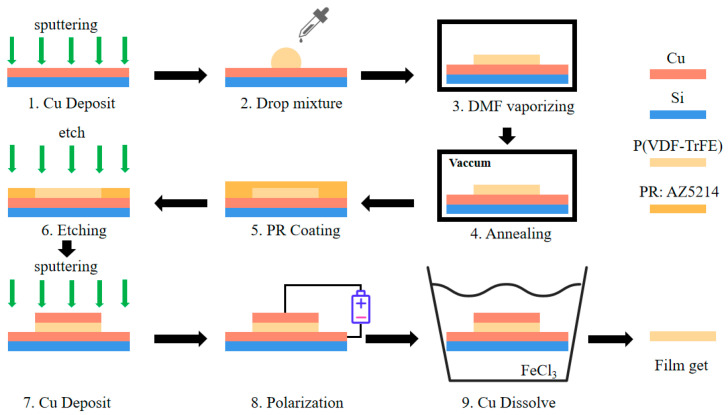
Fabrication process for P(VDF-TrFE) film.

The fabrication process of the P(VDF-TrFE) film is shown in Figure 1. Isotropic P(VDF-TrFE) film is firstly prepared by a solvent evaporation approach. P(VDF-TrFE) powder is dissolved in dimethylformamide (DMF) at a volume ratio of 1:10. Magnetic stirring at room temperature for at least 3 h is used to ensure the complete dissolution of P(VDF-TrFE) in DMF. Then, 100 µL of the P(VDF-TrFE) solution is applied dropwise on a 200 nm copper (Cu) film with silicon wafer as the substrate and placed in an oven at 60 °C for 8 h to vaporize the DMF solvent. The Cu layer is deposited by magnetron sputtering.

The second step is the recrystallization of the P(VDF-TrFE) film to obtain the high β-phase, low porosity, and low surface roughness. Two-step vacuum rapid annealing is used for the recrystallization, with the first step at 180 °C for 20 min and the second step at 150 °C for 1 h (sample S1). Two control groups are also made by one-step annealing. Their annealing parameters are 180 °C for 80 min (sample S2) and 150 °C for 80 min (sample S3), respectively. After cooling down to room temperature, a layer of AZ5214 photoresist is spin-coated on top of the P(VDF-TrFE) films and baked at 60 °C for 1 h. The films are then etched by ICP oxygen plasma to reduce its surface roughness. The experiments show that the photoresist and P(VDF-TrFE) have the same etch rate of 520 nm/min. Since the etching rates are the same for both polymers, the etching process removes the photoresist and the uneven part of the P(VDF-TrFE) film, which results in a smooth surface. Subsequently, a 200-nm Cu layer is deposited by magnetron sputtering on the upper surface of the films for polarization. DC poling over the film is performed at room temperature for 1 h with a 250 kV/mm electric field. The sample is then placed in FeCl_3_ solution to dissolve the Cu layers to obtain the floating P(VDF-TrFE) film.

### 2.2. Design of the P(VDF-TrFE) FBAR Pressure Sensor

**Figure 2:**
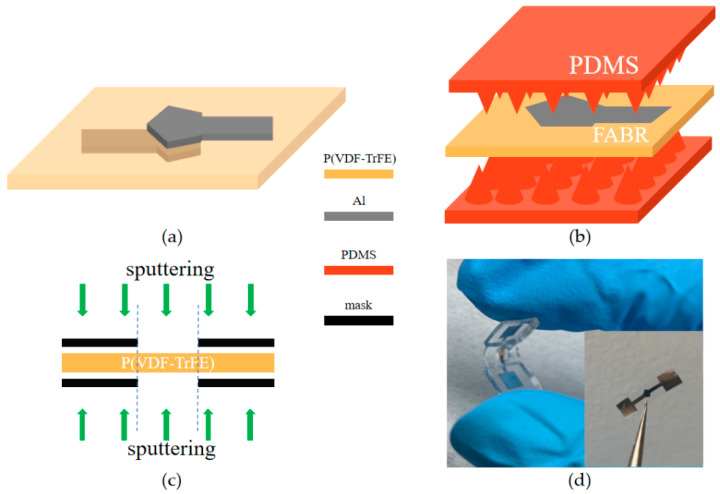
(**a**) Structural schematic of the P(VDF-TrFE) FBAR; (**b**) the exploded schematic diagram of the flexible pressure sensor; (**c**) electrode fabrication process for the FBAR; (**d**) optical photo of fabricated FBAR device demonstrating its flexibility.

Figure 2a shows a structural schematic of the P(VDF-TrFE) FBAR. The thickness of the P(VDF-TrFE) film is 30 μm, as this thickness leads to good mechanical strength while ensuring good transparency and flexibility. Aluminum electrodes with 200-nm thickness on both sides of the P(VDF-TrFE) film are used for the excitation of bulk acoustic waves. As the thickness of the aluminum electrodes is negligible, the resonant frequency of the device is determined by the following equation:

(1) $$f=\frac{c}{2d}$$

where c is the longitudinal sound velocity of the piezoelectric layer and d is the thickness of the piezoelectric layer.

Figure 2b shows the exploded schematic diagram of the flexible pressure sensor based on the P(VDF-TrFE). It consists of an FBAR sandwiched by two PDMS patches with microneedle arrays. For FBAR devices, the acoustic impedance mismatch between the upper and lower surfaces is the cause of acoustic reflection at the interface. The reflectivity R is determined by the following equation [24]:

(2) $${R=(Z}_{1}{-Z}_{2}{)/(Z}_{1}{+Z}_{2})$$

where Z_1_ and Z_2_ are the acoustic impedances of the incident layer and the target layer, respectively. The external pressure variation leads to the contact area change between the FBAR and the PDMS microneedles, which leads to a shift in the FBAR resonant characteristics. The size of the array is 5 × 5. Moreover, as the acoustic impedance of air is almost zero, the acoustic wave reflection at the contact point is much lower than that of the interface between P(VDF-TrFE) and air from Equation (2). In other words, part of the acoustic wave is transmitted to the PDMS and attenuated in it, which results in the weakening of the resonance amplitude. The variation in the resonance frequency and amplitude can be measured by its return loss (S_11_) using a network analyzer, which can be tracked for monitoring the pressure.

### 2.3. Fabrication of the P(VDF-TrFE) FBAR Pressure Sensor

As P(VDF-TrFE) can be attacked by acetone [25], it is difficult to fabricate P(VDF-TrFE) FBARs using conventional lithography processes which require acetone for photoresist removal. We thus use shadow masks for the fabrication of the top and bottom electrodes of the FBARs. As shown in Figure 2c, the hard masks block the sputtered aluminum outside the electrode area and only allow them to reach the desired electrode area. The shadow-mask-based process not only reduces the device fabrication cost, but also avoids the damage to the film properties caused by photolithography and other processes. Other fabrication methods, such as printed circuit board (PCB) technology [26,27], screen printing [28,29,30], and 3D printing technology [31] can also be applied to the fabrication of flexible devices. However, they are less suitable for the fabrication of the flexible FBARs presented in this paper. The electrode thicknesses made by PCB technology or screen printing are usually tens of microns, which are too thick for the flexible FBARs. In addition, the surface roughness of electrodes made by all three methods is much larger than that made by sputtering, which will significantly degrade the performance of the FBAR devices. Figure 2d shows an image of the fabricated P(VDF-TrFE) FBAR. The PDMS microneedle patch is made by a complementary silicon mold. The patch is attached to both sides of the FBAR device with the attitude of the needle tip touching the electrode of the FABR device. The upper and lower patches are bonded at the edges by PDMS.

### 2.4. Characterization Setup

The obtained P(VDF-TrFE) film is characterized in terms of morphology, crystalline phase, and piezoelectric coefficients using equipment including scanning electron microscopy (SEM, HITACHI SU5000, Tokyo, Japan), atomic force microscope (AFM, Bruker Dimension Icon, Karlsruhe, Germany), X-Ray diffraction (XRD, Shimadzu XRD-6100, Kyoto, Japan), Fourier transform infrared spectroscopy (FTIR, ThermoFisher NicoletiS50, Waltham, USA), and piezo-response force microscopy (PFM, Bruker Dimension Icon’s piezoelectric module, Karlsruhe, Germany). The return loss of the FBAR is measured by a network analyzer (Agilent E5071C, Santa Clara, USA), which is controlled by a PC. A MATLAB-based program is developed on the PC to implement automated measurements to record changes in the return loss of the FBAR devices. For heartbeat and respiration rate monitoring, we attach the device to a flexible PCB and then fix the PCB to a band. The band is fixed to the body in different ways to achieve different functions, which will be explained in detail in the Results and Discussion section.

## 3. Results and Discussion

### 3.1. Characterization of the P(VDF-TrFE) Films

#### 3.1.1. Morphological Features

Three different annealing temperature are used to treat P(VDF-TrFE) films, as aforementioned, to optimize the properties of the films.

**Figure 3:**
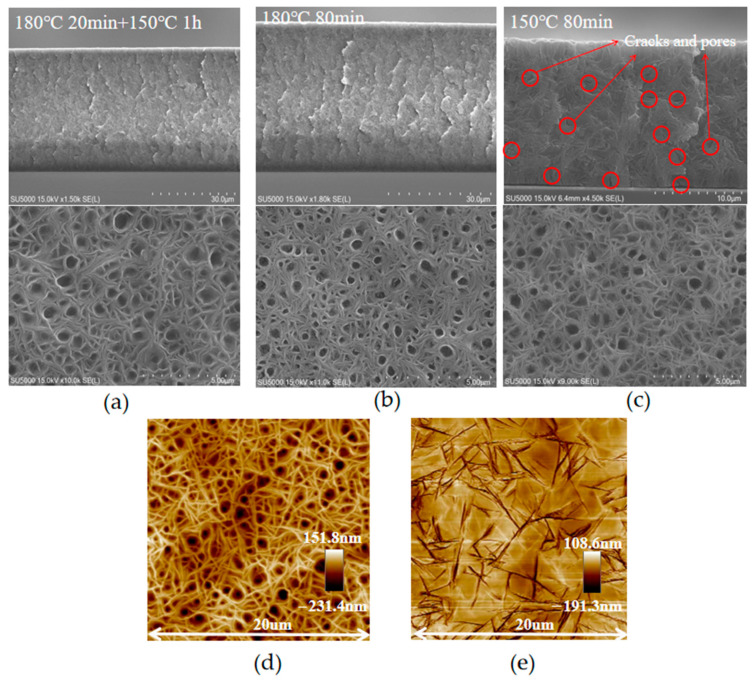
Cross-section and surface images of P(VDF-TrFE) film: (**a**) annealing temperature reaches above the melting point (sample S1); (**b**) annealing temperature always above the melting point (sample S2); (**c**) annealing temperature consistently below the melting point (sample S3), red circles mark the cracks and pores; (**d**) AFM image of the surface before etching treatment; (**e**) AFM image of the surface after etching treatment.

Figure 3 shows the SEM images of the P(VDF-TrFE) films treated at different annealing temperatures, which reveals their different morphological structures. Compared to sample S3 (Figure 3c, annealing at 150 °C for 80 min), sample S1 (Figure 3a, annealing at 180 °C for 20 min and 150 °C for 1 h) and sample S2 (Figure 3b, annealing at 180 °C for 80 min) have smaller grain sizes and denser structures. This is because annealing above the melting point changes the chain mobility and facilitates the polymer chains to align vertically on the substrate [32], which leads to a denser morphology and a smaller grain size. The internal cavities and cracks of sample S1 and sample S3 are also completely removed. This is because the flowability of the P(VDF-TrFE) film above the melting point facilitates the releasing of its internal air bubbles. However, due to the precipitation of air inside the melting film in the vacuum, a large number of circular pits appear on the surfaces of the films, which significantly increased the roughness of the films. This problem is solved by the photoresist-assisted ICP etching process. As shown by the AFM images in Figure 3d–e, the surface roughness is reduced from 70 nm to 40 nm before and after ICP etching.

#### 3.1.2. Crystalline Phases and Degree of Crystallinity

**Figure 4:**
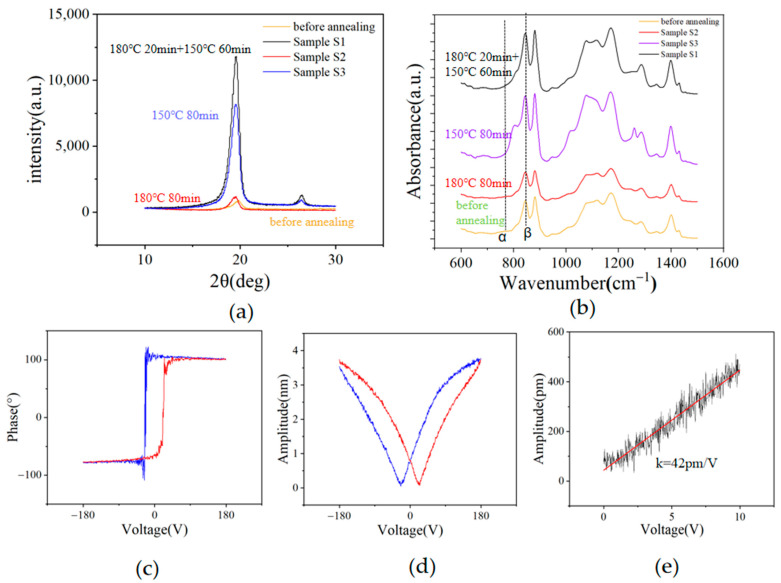
(**a**) XRD spectra; (**b**) FTIR spectra of the films with different annealing conditions; (**c**) phase and (**d**) amplitude curves of the piezoelectric response of the low-porosity P(VDF-TrFE) films under flip voltage; (**e**) the amplitude versus voltage curve when the voltage is less than the flip voltage, whose slope represents a d_33_ of 42 pm/V.

To characterize the crystallinity of the P(VDF-TrFE) films, we measured their XRD spectra, as shown in Figure 4a. The results show that sample S1 has the highest crystallinity. The crystallinity of sample S2 annealed above the melting temperature for 80 min is the lowest, with almost no improvement compared to the unannealed films.

Figure 4b shows the FTIR spectra of the P(VDF-TrFE) films. The electroactive and polar β-phase is identified by the specific absorption band at 844 cm^−1^, while there is no evidence of the nonpolar α-phase, which has a specific absorption band at 763 cm^−1^ [33]. FTIR spectra reflect the crystalline phase contents of materials. Figure 4b reveals that both sample S1 and S3 have higher β-phase contents than that of the unannealed film, while the β-phase content of sample S2 becomes lower after annealing.

P(VDF-TrFE) films are typically annealed between the Curie and melting temperatures. This is because when the ferroelectric material is annealed at a temperature higher than the Curie temperature, it changes from the ferroelectric phase into the paraelectric phase. The chain mobility of P(VDF-TrFE) is higher in the paraelectric phase compared to the ferroelectric phase, which facilitates the generation of all-trans conformation and results in a higher crystallinity. In addition, the chain mobility increases with the increasing temperature [34]. The XRD and FTIR spectra reveal that P(VDF-TrFE) films can hardly complete the crystallization at temperatures higher than the melting point, even with high chain mobilities. Nevertheless, annealing at temperatures above the melting point does not cause irreversible damage to the crystallization properties of the films. The films can thus still be annealed at temperatures below the melting point to complete the crystallization of the β-phase. Sample S1 thus has not only a lower porosity but also a higher crystallinity compared to sample S3.

#### 3.1.3. Electrical and Piezoelectric Properties

To obtain piezoelectricity, P(VDF-TrFE) films need to be polarized with an electric field more than twice their coercivity field (50 kV/mm) [35]. Because the breakdown strength of air is only 3 kV/mm, which is much smaller than the required 100 kV/mm, the applied electric field during polarization for films containing cavities may not reach 100 kV/mm, due to the air breakdown. The films containing cavities thus may not be properly polarized. In the experiments, it was found that the breakdown electrical field of sample S3 is between 50–100 kV/mm, which leads to an unstable polarization. However, sample S1 can be stably polarized under a 250 kV/mm electric field and remains intact under electric fields up to 300 kV/mm. This allows for the effective polarization for the film. The polarization direction of ferroelectrics can be changed under an electric field [36].

The PFM phase diagram in Figure 4c shows that a phase difference of 180° is observed in both directions of the voltage sweeping loop. This means that the P(VDF-TrFE) film has good ferroelectricity and can be polarized to obtain piezoelectricity [37]. The complete butterfly circuit (shown in Figure 4d) under the reversal of bias voltage further confirms the piezoelectricity of the film. For the film after polarization, we applied a bias voltage smaller than the flip voltage at four random points on the film and obtained the piezoelectric coupling coefficient (d33) from the slope of the amplitude vs. the voltage curve. The piezoelectricity of the four points is not uniform. The amplitude curve shown in Figure 4e yields the highest d33 value among the four points, with a value of 42 pm/V. The amplitude curves of the remaining three points are shown in Figure S1.

### 3.2. Device Characterization and Testing

#### 3.2.1. Electrical Characterization of the P(VDF-TrFE) FBAR

**Figure 5:**
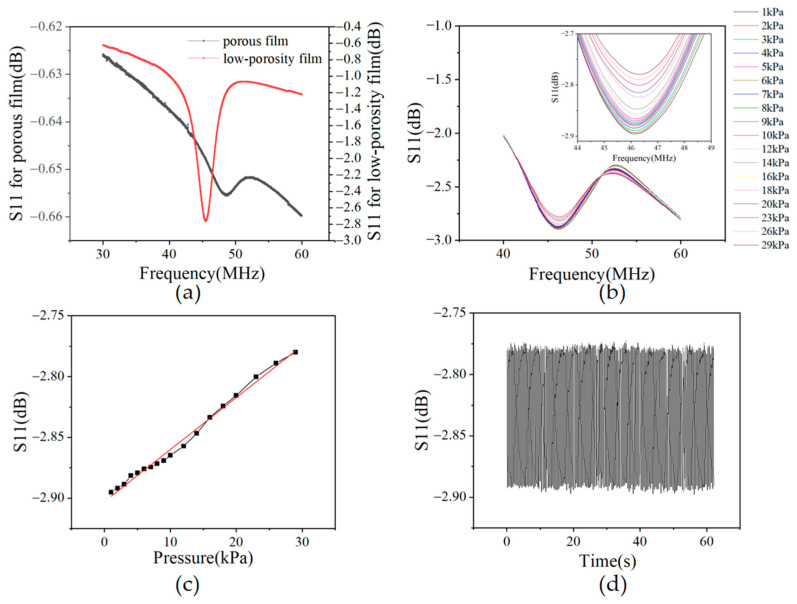
(**a**) Measured S_11_ of FBARs based on porous and low-porosity P(VDF-TrFE) films; (**b**) S_11_ of the device under different pressures; (**c**) the peak amplitude of S_11_ in response to pressure variation; (**d**) S_11_ peak amplitude response under cyclic pressure from 0 to 30 kPa for 150 cycles.

Figure 5a shows the measured S_11_ of the FBAR devices made of different films. The thickness and longitudinal acoustic wave velocity of the P(VDF-TrFE) film are 30 μm and 2200 m/s, respectively. Using Equation (1), the resonant frequency of the longitudinal mode of the FBAR device is calculated at 36.6 MHz. The test results are roughly in line with the calculated value. The slight deviation is due to uneven film thickness. The peak sharpness in the S_11_ curve represents the resonance strength of the FBAR. Figure 5a shows that the peak of the FBAR based on low-porosity P(VDF-TrFE) film is much sharper than the FBAR based on films utilizing conventional processes, which indicates the higher resonance performance and lower acoustic loss of the low-porosity P(VDF-TrFE) film. Although there is a considerable improvement in the resonance performance, it still does not meet the standard of wireless passive testing, which requires us to further improve the film quality through further work in the follow-up.

#### 3.2.2. Pressure Sensing Characteristics of the P(VDF-TrFE) FBAR

The performance of the flexible pressure sensor consisting of the P(VDF-TrFE) FBAR and PDMS patches is investigated by a compression test setup. A robotic arm is used to apply pressure to the device with a pressing area larger than the PDMS patch. The pressure changes are monitored by recording the peak value (minimum) variation of the S_11_ curve on the network analyzer. Figure 5b shows the S_11_ curves of the device under different pressures. The device performs well over a pressure range of up to 30 kPa. The peak S_11_ value of the sensor exhibits a linear response to the pressure, as shown in Figure 5c. A cyclic pressure test with unique loading in each cycle is performed to verify the repeatability of the device. The number of test cycles is 150, with pressure varying from 0 to 30 kPa. As shown in in Figure 5d, the average S_11_ peak response is −2.77 ~ −2.89 dB when the pressure varies from 0 to 30 kPa. The small variation of the sensor response reveals it excellent stability and favorable fatigue properties. The tensile strength of the sensor is determined by a stretch test. Figure S2 shows the experimental setup. The test results show that the modulus of the film is 0.427 GPa. The maximum strain that the film can withstand before fracturing is 15.2%. When the film breaks, the electrode still remains intact. The maximum stretchability before the failure of the device is thus 15.2%.

The pressure sensitivity of the device is related to the size, shape, and softness of the PDMS microneedle. Theoretically, the pressure sensitivity increases with the increase in the tip size and softness of the PDMS microneedle. In contrast, its dynamic range increases with the decrease in the tip size and softness. This allows for the tuning of the sensitivity and dynamic range of the sensor by adjusting the size and preparation parameters of PDMS microneedles.

#### 3.2.3. Heart Rate and Breathing Monitoring

**Figure 6:**
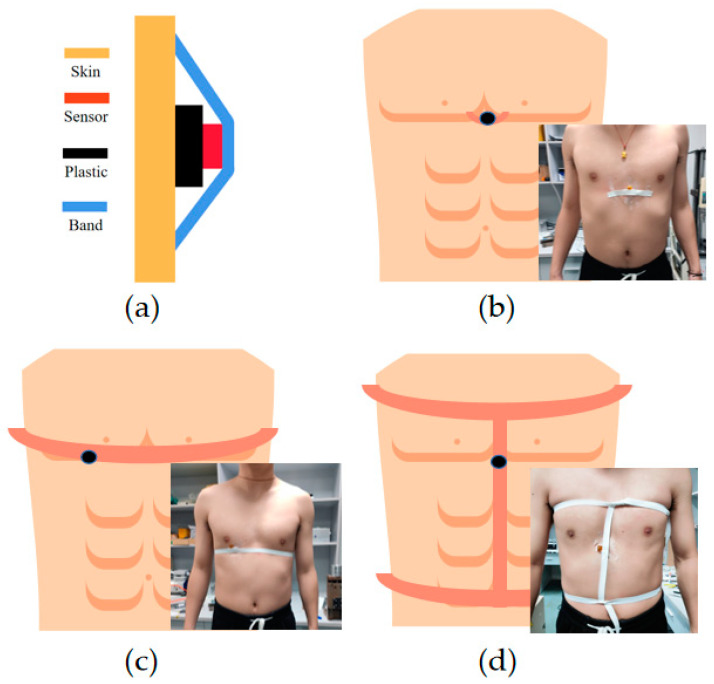
(**a**) Fixation structure of the pressure sensor for vital signal monitoring; (**b**) device fixation method for heartbeat monitoring; (**c**) device fixation method for breathing monitoring; (**c**) device fixation method for simultaneous monitoring of heart rate and respiratory rate.

The pressure sensor is constructed by sandwiching the P(VDF-TrFE) FBAR with two PDMS microneedle patches. It is attached to the skin surface of human body for heart rate and breathing monitoring, as shown in Figure 6a. A plastic cylinder with a bottom area larger than the PDMS patch is attached to the skin with medical tape. The pressure sensor is soldered to a flexible PCB that is fixed to an elastic band. The two ends of the elastic band are taped to the skin surface so that the sensor is in tight contact with the plastic cylinder. The plastic cylinder vibrates with the skin and compresses the PMDS patch that produces the sensor response. The pressure responses are monitored by recording the peak value (minimum) variation of the S_11_ curve on the network analyzer. An image of the test system is shown in Figure S3.

**Figure 7:**
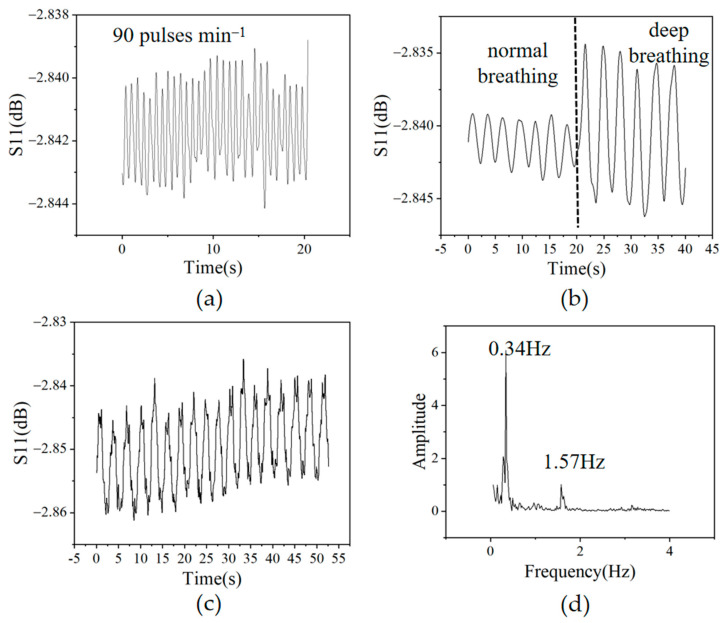
(**a**) Heartbeat monitoring result shows that the volunteer’s heart rate is 90 pulses min^−1^ with (**b**) time-domain response of the sensor including both normal breathing and deep breathing; (**c**) time-domain fusion response of the sensor to heartbeat and breath; (**d**) frequency spectra of the fusion response showing a heartbeat frequency of 1.57 Hz and respiration frequency of 0.34 Hz.

Different sensor fixing methods are used to accomplish the measurement of different physiological signals. To achieve the detection of the heartbeat, the sensor is attached to the heart position of the chest, as shown in Figure 6b. The skin above the heart vibrates during the heartbeat, which is conducted to the PDMS patch of the pressure sensor through the plastic cylinder. As shown in Figure 7a, the peak S_11_ value of the sensor exhibits a response that follows the heartbeat of the volunteer. The heart rate measured by the P(VDF-TrFE) FBAR pressure sensor is 90 min^−1^, which agrees with the manual pulse recording results. The fast heart rate of the volunteer is due to the fact that the test is completed with the volunteer’s clothes taken off at a relatively low laboratory temperature in the winter and the volunteer needs a fast heart rate to maintain his body temperature. As can be seen from the results, the pressure sensor can well track the heartbeat of the volunteer.

Figure 6c shows the sensor fixation method for respiration rate monitoring. The cylindrical plastic is attached to the non-heartbeat region of the right chest, while the elastic band encircles the body. The expansion and contraction of the chest in the respiration cycle changes the contact pressure between the band and the sensor, which results in a sensor response following the respiration cycle. The magnitude of the response is related to the breath depth. Figure 7b shows the time-domain sensor response, in which the normal breathing and deep breathing cycles can be clearly distinguished by their response amplitudes. The results indicate that the device can track both the respiration rate and intensity.

The diagnosis and in-patient monitoring of many diseases require the simultaneous detection of both heart rates and respiratory rates [38]. This can be achieved by our pressure sensor using the fixation method shown in Figure 6d. In this fixation, the relative motion of the plastic cylinder to the device is influenced by both breathing and heartbeat. Figure 7c shows the time-domain response of the fusion monitoring signal. It can be seen that two signals of different frequencies can be observed in the response curve. The small amplitude heartbeat signal is modulated on the large amplitude breath vibration signal. Figure 7d shows the frequency spectra of the fusion response signal. The 0.34 Hz and 1.57 Hz frequency components correspond to the respiration and heartbeat frequencies, respectively. The clear separation and large signal-to-noise ratio of the two peaks in the frequency spectra reveals the sensor’s ability in the precise simultaneous tracking of both respiration and heart rates.

The above results show that the FBAR pressure sensor based on low-porosity P(VDF-TrFE) film can achieve the separate or simultaneous monitoring of human respiration and heart rates. In recent decades, various pressure sensors have been developed for heartbeat and respiration monitoring. Table 1 shows a comparison between the different techniques. Compared to previous works, our sensor has the advantages of good linearity, fast response, low temperature drift, low cost, and dual-mode detection capability.

## 4. Conclusions

In summary, we proposed a novel process based on two-step annealing for the fabrication of high-quality P(VDF-TrFE) films. The two-step annealing included one annealing above the melting point (180 °C for 20 min) to allow the release of internal air bubbles and one annealing below the melting point (150 °C for 60 min) for the recrystallization of the β-phase. The obtained P(VDF-TrFE) film exhibited a low porosity, low surface roughness, and high piezoelectric coupling coefficient (d_33_ = 42 pm/V). The flexible FBAR device fabricated using the low-porosity P(VDF-TrFE) film achieved strong resonance with a sharp S_11_ peak. A pressure sensor was made by sandwiching the P(VDF-TrFE) FBAR with two PDMS microneedle patches. Separate and simultaneous monitoring of respiration and heart rates were demonstrated using the developed pressure sensor under different fixation setups. The low-porosity P(VDF-TrFE) film and the FBAR device reported in this work demonstrated the feasibility of flexible piezoelectric acoustic resonators, which could see wide applications in wearable sensors for both physical and chemical sensing.

## Figures and Tables

**Table 1:** Comparison between various pressure sensors for heart rate and respiration monitoring.

Sensor Type	Sensitivity	Linearity	Response Time	Monitoring Modes	Stretcha-bility	Cost	Temperature Drift
Piezoresistive Pressure [23]	0.075 kPa^−1^ $(\Delta R/R_{0}$)/kPa	Low	120 ms	Heartbeat	-	High	Medium
Piezoelectric Strain [39]	0.97 mV/με	-	-	Heartbeat and breath	35%	Low	High
Triboelectric Pressure [40]	18.98 V/kPa	Low	-	Heartbeat and breath	-	Med-ium	-
Capacitive pressure [22]	7.847 kPa^−1^ $(\Delta C/C_{0}$)/kPa	Medium	20 ms	Pulse	-	High	Low
This work	0.004 dB/kPa	High	25 ms	Heartbeat and breath	15%	Low	Low

## Data Availability

The data presented in this study are available on request from the corresponding author.

## Supplementary Materials

The following supporting information can be downloaded at: https://www.mdpi.com/article/10.3390/s23042136/s1, Figure S1: The amplitude vs voltage curves of three other PFM testing points on the PVDF-TrFE film; Figure S2: Device stretchability test setup; Figure S3: Image of the test system setup.
