# Supplementary material for: Flexible Film Bulk Acoustic Resonator Based on Low-Porosity β-Phase P(VDF-TrFE) Film for Human Vital Signs Monitoring
Source: Sensors (Basel). 2023 Feb 14;23(4):2136. doi: 10.3390/s23042136 (PMC9962757; doi:10.3390/s23042136)
Supplement: Supplementary file 1 [file sensors-23-02136-s001.zip › sensors-2192924-supplementary.pdf]

Supplementary Materials

Flexible Film Bulk Acoustic Resonator based on Low-porosity β-phase P(VDF-TrFE) Film for Human Vital Signs Monitoring

Zhentao Yu [1], Feng Gao [1,2,*], Xiangyu He[1], Hao Jin[1,2], Shurong Dong[1,2], Zhen Cao[1], Jikui Luo[1,2]

1. Key Lab. of Advanced Micro/Nano Electronic Devices and Smart Systems of Zhejiang, College of Information Science and Electronic Engineering, Zhejiang University, Hangzhou 310027, China;
2. ZJU-Hangzhou Global Scientific and Technological Innovation Center, Hangzhou 311215, China;

* Correspondence: gao.feng@zju.edu.cn (F.G.);

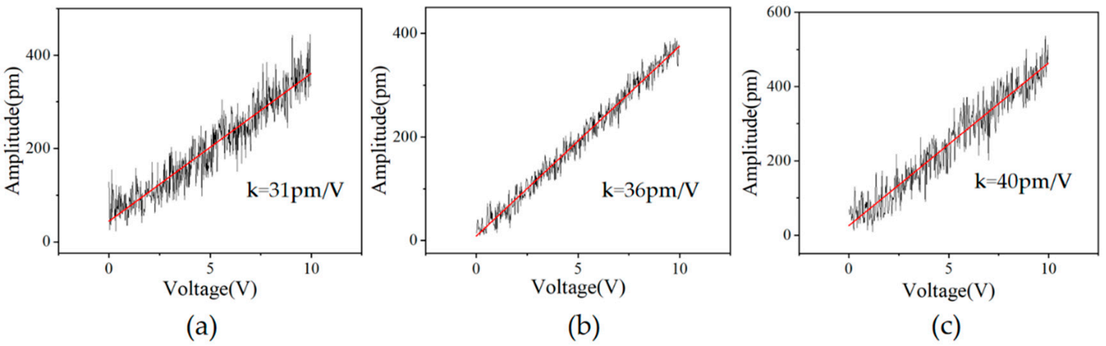

Figure S1. The amplitude vs voltage curves of three other PFM testing points on the PVDF-TrFE film.

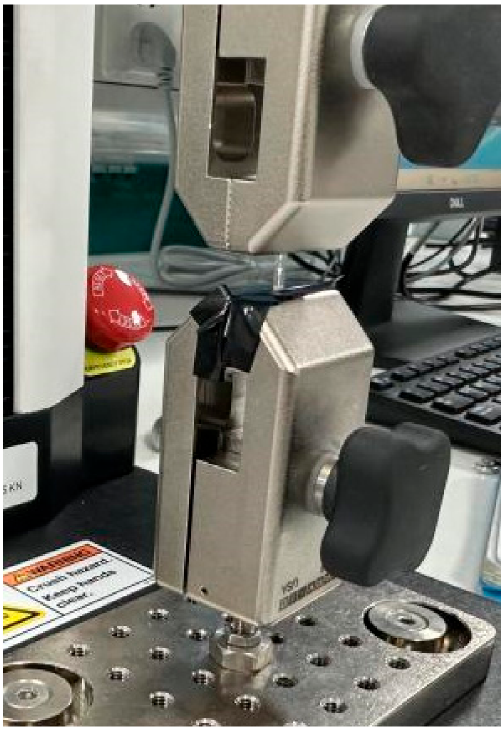

Figure S2. Device stretchability test setup.

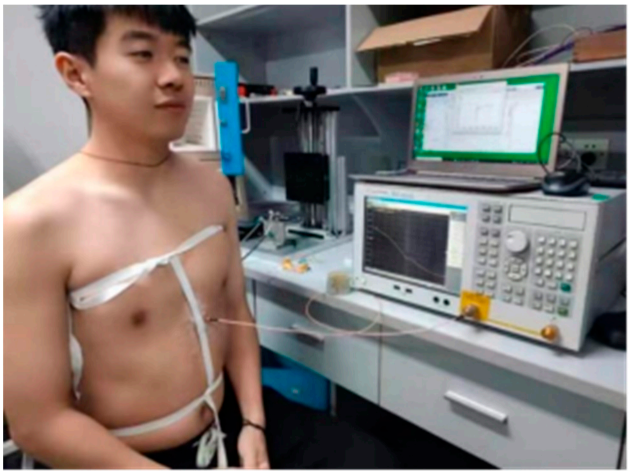

Figure S3. Image of the test system setup.
